# News and Miscellany

**Published:** 1902-09

**Authors:** 


### News and Miscellany.

---

**McGill University** proposes to inaugurate a five years' course in medicine.

---

**A woman,** Dr. Rina Mastio, has been appointed to the professorship of anatomy at the University of Milan.

**Of the 402 candidates** who were recently examined by the Pennsylvania Board of Medical Examiners 352 passed.

---

**The New Texas Medical Practice Law,** in pamphlet, 20 cents in stamps. TEXAS MEDICAL JOURNAL, Austin, Texas.

---

**An International Congress** in tuberculosis will be held in Berlin in October. Two delegates from each country are invited.

---

**The Charity Hospital** at Shreveport, La., is to be improved by the expenditure of $35,000, appropriated by the State for that purpose.

---

**The Albany (New York) Hospital** is building a $40,000 pavilion for contagious diseases, the money for which has been voted by the city council.

---

**Dr. Clarence Warfield,** one of San Antonio's most successful specialists on diseases of the Eye, Ear, Nose and Throat, is spending three months in post-graduate study at Berlin and Paris.

---

**The Litchfield County** (Connecticut) Medical Society, founded in 1765, is still in existence, and is in a flourishing condition. This is said to be the oldest medical society in the country.

---

**Dr. and Mrs. C. R. Myrick** and Miss Myrick are spending the summer in Boerne. The doctor has been devoting a great deal of time lately to preparing his fast bay gelding, Bucephalus, to enter the races at San Antonio in October.

---

**The Pennsylvania** State Medical Society will hold its fifty-second annual meeting at Allentown on September 16-18. Addresses will be delivered by G. D. Nutt, J. M. Baldy, A. O. J. Kelly, J. M. Murdoch and G. H. Halberstadt.

---

**Active steps are** being taken in London by the Sanitary Committee of that city to abate the spitting nuisance. Circulars are being issued, and telegraph, railway and omnibus companies, post offices, etc., are being asked to co-operate with the committee.

---

**He was sitting** by her side at dinner, proudly congratulating himself upon being where he could look down upon the beautiful neck and arms. "I am being tortured," she said, as she moved uneasy. "I have been vaccinated, and it is just 'taking.'" "Why," he said, unguardedly, as he cast another glance at that handsome neck and those lovely arms, "where were you vaccinated?" "In Boston," she replied, as a smile drove away the evidences of pain.

**A young man** calling on his sweetheart for the first time since her return from abroad, inquired about the pleasures of her sea voyage. "Were you sick both ways?" he asked, solicitously. "No; I only vomited," she demurely replied.—*Southern Clinic.*

---

**Married** in Austin, August 11 (ult.), Dr. R. H. Eanes of Taylor, to Miss Florence Hill of Austin. Dr. and Mrs. Eanes are in Colorado on their honeymoon. The JOURNAL extends its congratulations to its friend Eanes on his capture of so splendid a prize.

---

**Dr. H. K. Leake** of Dallas, Texas, is on a visit to New York and other eastern cities. He will attend the meeting of the Association of American Obstetricians and Gynecologists, to be held at Washington September 16th, and will return home about September 25th.

---

**The New York Medical Record** reports the case of a woman in Valencia, 44 years old, who has during her twenty-four years of married life given birth to twenty-four children, all born alive and at term, and all single births. One physician has assisted at the delivery of nineteen of these.

---

**The New York** State Hospital for Consumptives, started under a $100,000 appropriation made by the Legislature, will be opened next year. It is to be a two story brick and stone structure built on the pavilion plan, and will accommodate one hundred patients. It is to be located in the Adirondacks.

---

**The West Texas** Medical Association will hold its twenty-seventh annual meeting in San Antonio during the last week in October. The membership of this society has increased eighty per cent. during the past year, and the average attendance at the meetings has increased nearly four hundred per cent.

---

**San Antonio's** new fireproof sanitarium, to be known as "The Physicians and Surgeons Hospital," has now been in course of construction for six weeks. The institution will be made absolutely proof against fire, and will be one of the best arranged and best equipped private hospitals in the South. There are thirty-four physicians among the stockholders.

---

**The death rate statistics** for 1901 show that during last year Iowa was the healthiest State, with a death rate of 9.2 per 1,000, and that Louisiana made the poorest showing with a death rate of 20.65 per 1,000. Among the largest cities the five with the highest rates were as follows: Charleston 29.11, New Orleans 21.44, Washington 21.14, Baltimore 20.25, New York 20. Chicago had the lowest, 13.88.

**Dr. S. E. Hudson** of Austin, formerly Associate Editor and Manager of the "Red Back," is in New York attending the polyclinics and hospitals. Dr. Hudson will spend the months of September and October in this post-graduate study. I commend him most cordially to the profession, and bespeak for him every courtesy at the hands of my friends and friends of the "Red Back."

---

**Practice for Sale.**—Residence and practice worth $2,500 to $4,000 in a rich farming country settled with thrifty Bohemian and Germans, in a railroad town of 1600 in S. W. Texas, can be bought for $2,000, half cash. Will stay with purchaser till December 31, and induct him into the practice. Seller desires to go to a city. Address W. R. E., care TEXAS MEDICAL JOURNAL, Austin, Texas.

---

**New Orleans Polyclinic.**—Sixneenth annual session opens November 3, 1902, and closes May 30, 1903. Physicians will find the Polyclinic an excellent means for posting themselves upon modern progress in all branches of medicine and surgery. The specialties are fully taught, including laboratory work. For further information address NEW ORLEANS POLYCLINIC, Postoffice Box 797, New Orleans, La.

---

**The "Swearingen."**—It has been announced by State Health Officer Tabor that he had decided to name the new quarantine disinfecting vessel at the port ot Galveston "Swearingen," in honor of the memory of Dr. R. M. Swearingen, long time State health officer of Texas.

The new vessel is now in course of construction at Philadelphia and will be ready for use this fall. The ship will cost $37,250, although $45,000 was appropriated for that purpose by the last legislature.

---

**The Red Back.**—SPECIMEN LETTER.

ALBANY, TEX., July 9, 1902.

*Dear Doctor Daniel:*

Inclosed you will please find Bank M. O. for two dollars ($2.00) to pay for RED BACK, one of the best journals that comes to me. Living as I do in the grasshopper and drouth ridden district, all of my last year's work so far is a clear loss. Still I *can not do without the* RED BACK.

Very truly yours,

W. M. POWELL.

(Renews for eighteenth consecutive year.—D.)

---

*To Our Medical Friends and Patrons:*

We respectfully announce Drs. Holland, Jelks and Bernart, successors to Drs. Jelks and Holland.

Dr. F. W. Jelks is a son of the late Dr. J. T. Jelks, and has been for ten years in hospital and private practice in St. Louis.

Dr. Wm. F. Bernart has been practicing here for several years, and for two years A. A. Surgeon at the Army and Navy Hospital, this city.

All medical and surgical work will be taken care of as formerly. Offices will remain in Thompson Building, 218 Central Avenue. The new firm will constitute the medical and surgical staff of the Ozark Sanatorium, and managers and editors of the *Hot Springs Medical Journal.*

It is our hope to continue to merit the confidence reposed in the firm of Jelks & Holland.

Sincerely,

DRS. HOLLAND, JELKS & BERNART.

Hot Springs, Ark., Aug. 8, 1902.

